# Loss of Lipid Virulence Factors Reduces the Efficacy of the BCG Vaccine

**DOI:** 10.1038/srep29076

**Published:** 2016-06-30

**Authors:** Vanessa Tran, Sang Kyun Ahn, Mark Ng, Ming Li, Jun Liu

**Affiliations:** 1 Department of Molecular Genetics, University of Toronto, Toronto, ON, Canada

## Abstract

Bacille Calmette-Guérin (BCG), an attenuated strain of *Mycobacterium bovis*, is the only vaccine available for tuberculosis (TB) control. BCG comprises a number of substrains that exhibit genetic and biochemical differences. Whether and how these differences affect BCG efficacy remain unknown. Compared to other BCG strains, BCG-Japan, -Moreau, and -Glaxo are defective in the production of phthiocerol dimycocerosates (PDIMs) and phenolic glycolipids (PGLs), two lipid virulence factors. To determine if the loss of PDIMs/PGLs affects BCG efficacy, we constructed a PDIM/PGL-deficient strain of BCG-Pasteur by deleting *fadD28*, and compared virulence, immunogenicity, and protective efficacy in animal models. SCID mouse infection experiments showed that ∆*fadD28* was more attenuated than wild type (WT). The ∆*fadD28* and WT strains induced equivalent levels of antigen specific IFN-γ by CD4^+^ and CD8^+^ T cells; however, ∆*fadD28* was less effective against *Mycobacterium tuberculosis* challenge in both BALB/c mice and guinea pigs. These results indicate that the loss of PIDMs/PGLs reduces the virulence and protective efficacy of BCG. Since the loss of PDIMs/PGLs occurs naturally in a subset of BCG strains, it also suggests that these strains may have been over-attenuated, which compromises their effectiveness. Our finding has important implications for current BCG programs and future vaccine development.

Tuberculosis (TB) is a leading cause of death worldwide. According to a recent report by the World Health Organization (WHO), TB killed 1.5 million and caused illness in 9.6 million people in 2014. Bacille Calmette-Guérin (BCG) is the only licensed vaccine against TB. Although it is effective at reducing disseminated forms of TB (e.g., miliary TB and tubercular meningitis) in children^1^,^2^, BCG has highly variable efficacy (0–80%) against adult pulmonary TB^3^,^4^, the most contagious form of the disease. Another concern with BCG is its safety in immunocompromised individuals. Disseminated BCG disease has been observed in HIV-infected children following BCG vaccination^5^, and the risk outweighs the benefit of TB prevention^5^,^6^. In 2007, the WHO revised its recommendation and declared that HIV infection is a contraindication for giving BCG^7^. In light of this, there is an urgent need to develop a more effective and safe TB vaccine.

One hypothesis to account for the highly variable protective efficacy of BCG observed in clinical trials concerns the heterogeneity of BCG strains^8^. Although colloquially referred to as BCG, there are a number of BCG substrains that have been used in different vaccination programs^9^,^10^. Genetic differences including deletions and duplications of genomic regions and single nucleotide polymorphisms (SNPs) among these BCG strains have been well documented, based on a number of studies including whole genome sequencing^9^,^11^,^12^,^13^,^14^,^15^. As such, it was suggested that the strain variation may contribute to the variable efficacy of BCG and that some BCG strains might have been over-attenuated during the *in vitro* passaging and consequently lost effectiveness^16^. However, this hypothesis has not been formally tested due to the paucity of clinical studies directly comparing different BCG strains. In addition, although genetic and biochemical differences among BCG strains are well established^17^, whether and how these differences affect BCG effectiveness against TB are largely unknown and remain a matter of debate^8^,^18^.

Previously, we found that BCG-Japan, -Moreau, and -Glaxo are naturally deficient in the production of phthiocerol dimycocerosates (PDIMs) and phenolic glycolipids (PGLs), whereas the other nine BCG strains tested, including BCG-Pasteur, produced abundant levels of PDIMs and PGLs^19^. PDIMs and PGLs are structurally related complex lipids in the mycobacterial cell wall and are critical for mycobacterial virulence^20^. PDIMs are present only in pathogenic mycobacteria such as *Mycobacterium tuberculosis* (*M. tb*)*, M. bovis,* and *M. marinum*. PGLs are also restricted to pathogenic mycobacteria except that in *M. tb*, only a subset of clinical isolates produces PGLs^20^. PDIMs were first implicated in virulence using signature-tagged transposon mutagenesis which identified *M. tb* PDIM mutants that were attenuated in mice^21^,^22^. Since then, PDIMs have been shown to mediate receptor-dependent phagocytosis of *M. tb*^23^, and contribute to cell wall permeability^24^ and protection against bactericidal effects of reactive nitrogen intermediates in activated macrophages^25^. PGLs have been implicated in dampening the immune response by inhibiting the release of pro-inflammatory cytokines and have been associated with a hypervirulent phenotype of certain *M. tb* clinical isolates^26^,^27^. The critical role PDIMs/PGLs in virulence has also been demonstrated in *M. bovis*^28^ and *M. marinum*^29^. Recently, a study in *M. marinum* suggested that PDIMs and PGLs work in a concerted fashion to recruit permissive macrophages and restrict macrophages with high bactericidal activities, which favors mycobacterial survival and replication in the host^30^.

Given that PDIMs/PGLs play important roles in host-pathogen interactions, it is of great interest to determine whether the loss of PDIMs/PGLs, which occurs naturally in a subset of BCG-strains, affects BCG vaccine properties in terms of safety and protective efficacy. In this study, we constructed a PDIM/PGL-deficient strain of BCG-Pasteur by targeted deletion of *fadD28*, a biosynthetic gene of PDIMs/PGLs, and performed comparative analyses of virulence and protective efficacy of the isogenic strains. We found that the PDIM/PGL-deficient strain was less virulent than the wild type (WT) strain of BCG-Pasteur in SCID mice, but was also less protective against *M. tb* infection in both BALB/c mice and guinea pigs.

## Results

### Construction of an isogenic PDIM/PGL deficient mutant of BCG-Pasteur

**Figure 1:**
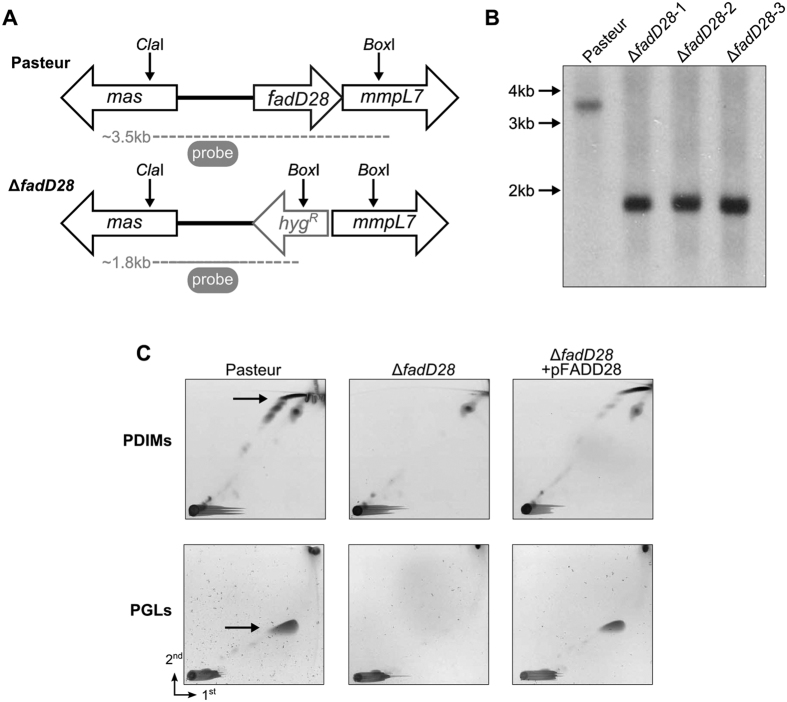
Construction of a PDIM/PGL deficient strain of BCG-Pasteur. (**A**) Genomic organization of WT (Pasteur) and ∆*fadD28* strains. Dashed lines indicate products of restriction digestion with *Cla*I and *Box*I. (**B**) Southern blot analysis. Chromosomal DNAs isolated from WT and three randomly picked ∆*fadD28* clones were digested with *Cla*I and *Box*I and blotted with a 500 bp probe of *fadD28*, which yielded a 3.5 kb and 1.8 kb fragment, respectively, and agreed with prediction (**A**). (**C**) 2D-TLC analysis of PDIMs and PGLs. For PDIM analysis, apolar lipids were developed with petroleum ether/ethyl acetate (98:2 v/v, 3 times) in the first dimension (1^st^) and petroleum ether/acetone (98:2, v/v) in the second dimension (2^nd^). Lipids were visualized by charring with 5% phosphomolybdic acid. For PGL analysis, the apolar lipid extract was developed with chloroform/methanol (96:4, v/v) and toluene-acetone (80:20, v/v), followed by charring with α-naphthol. PDIMs, phthiocerol dimycocerosates; PGLs, phenolic glycolipids.

A BCG-Pasteur strain deficient in PDIMs/PGLs was generated by target deletion of *fadD28* (Fig. 1A,B), which encodes a fatty acyl-AMP ligase involved in PDIM/PGL biosynthesis^20^. Deletion of *fadD28* was confirmed by Southern blot using a 500 bp probe against the upstream region of *fadD28* (Fig. 1A,B). The Δ*fadD28* strain grew equally well as the WT strain in 7H9 medium (Supplementary Fig. S1). Analysis of cell wall lipids by two-dimensional thin layer chromatography (2D-TLC) showed that Δ*fadD28* was defective in the synthesis of PDIMs/PGLs (Fig. 1C). Transformation of plasmid pFADD28, which contains intact *fadD28*, into the knockout strain restored the production of PDIMs and PGLs in the cell wall (Fig. 1C).

### Loss of PDIMs/PGLs reduces virulence of BCG-Pasteur

**Figure 2:**
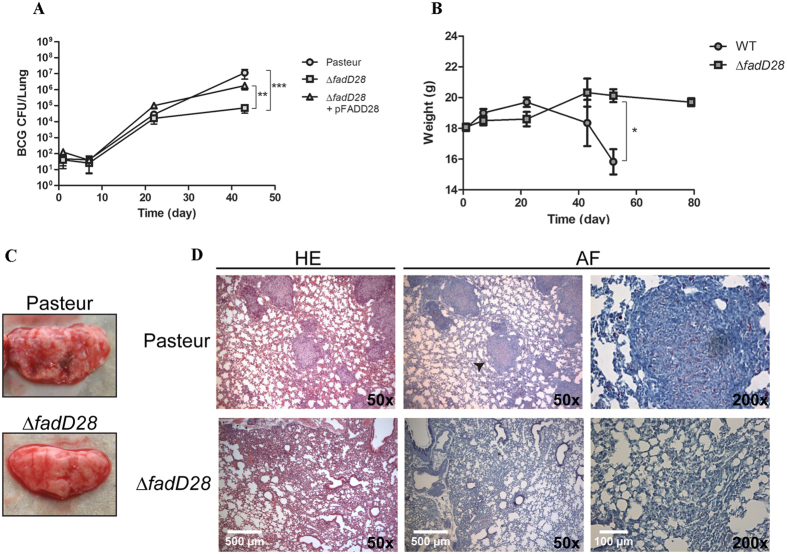
The PDIM/PGL deficient mutant of BCG-Pasteur is less virulent in SCID mice. (**A**) BCG burden in the lungs of infected SCID mice. SCID mice were infected intravenously with 10^4^ CFU of BCG-Pasteur (Pasteur), the ∆*fadD28*, or complemented strain (∆*fadD28* + pFADD28). Bacterial burden in the lungs were determined at various time points. (**p < 0.01; ***p < 0.001, two-way ANOVA). (**B**) Body weight of SCID mice infected with 10^5^ CFU of WT BCG-Pasteur or ∆*fadD28* (*p < 0.05; student’s *t*-test). (**C**) Lung pathology of mice infected with WT BCG-Pasteur and ∆*fadD28* (10^5^ CFU) at 52 day post infection. (**D**) Lung histology of mice infected with WT BCG-Pasteur and ∆*fadD28* (10^5^ CFU) at 52 day post-infection; arrow denotes magnified granuloma-like lesion. HE, hematoxylin-eosin stain; AF, acid fast stain.

Given the critical role of PDIMs/PGLs in pathogenic mycobacteria, the loss of PDIMs/PGLs in a BCG strain will likely reduce its virulence. On the other hand, since BCGs are already attenuated strains, the extent to which the additional loss of PDIMs/PGLs contributes to the attenuation of BCG remains unknown. To address this question, we compared the virulence of WT, ∆*fadD28*, and the complemented strains in severely immunocompromised SCID mice, a mouse model that has been commonly used to assess the safety of BCG strains including the recombinant BCG and attenuated *M. tb* vaccine candidates^31^,^32^,^33^. The safety of a live vaccine is inferred from its virulence in SCID mice, which is reflected in the ability of the vaccine to replicate in the animal and to cause mortality. Groups of SCID mice were infected intravenously via the tail vein with ~10^4^ colony forming units (CFU) of each strain. At 43 days post-infection (dpi), WT BCG-Pasteur reached an average of 7.05 log_10_ CFU in the lungs of SCID mice, whereas the ∆*fadD28* strain exhibited reduced growth during the same period, with an average of 4.85 log_10_ CFU in the lungs, which is 2.2 log_10_ CFU lower than WT (*p* < 0.001, two-way ANOVA, Fig. 2A). The complemented strain had on average 6.25 log_10_ CFUs in the lungs of SCID mice at the same time point, which was not significantly different to that of WT (Fig. 2A). A similar trend was observed for BCG counts in the spleen of SCID mice, although there was no difference between the ∆*fadD28* and the complemented strains (Supplementary Fig. S2).

In a separate experiment, SCID mice (4 mice per group) were infected with a higher dose (10^5^ CFU) of BCG strains (WT and ∆*fadD28*) and monitored for morbidity over time. WT-infected mice began to lose body weight at 22 dpi (Fig. 2B). By 52 dpi, these mice exhibited severe dehydration and weight loss (≥20%) and were euthanized to comply with our animal protocols. In contrast, none of the SCID mice infected with ∆*fadD28* exhibited significant weight loss or other disease phenotypes at 52 dpi and remained healthy until the experiment was terminated at 80 dpi (Fig. 2B). SCID mice infected with WT BCG-Pasteur also exhibited gross pathological evidence of disease, with numerous surface nodules observed in the lungs, whereas few were seen in the ∆*fadD28*-infected mice (Fig. 2C). Consistently, histological analysis of lung tissues from WT-infected mice had numerous acid-fast positive granulomatous lesions, whereas lungs from ∆*fadD28*-infected mice showed few scattered lesions (Fig. 2D). Taken together, our results demonstrate that loss of PDIMs/PGLs reduces the virulence of BCG-Pasteur.

### Loss of PDIMs/PGLs does not affect immunogenicity of BCG-Pasteur

**Figure 3:**
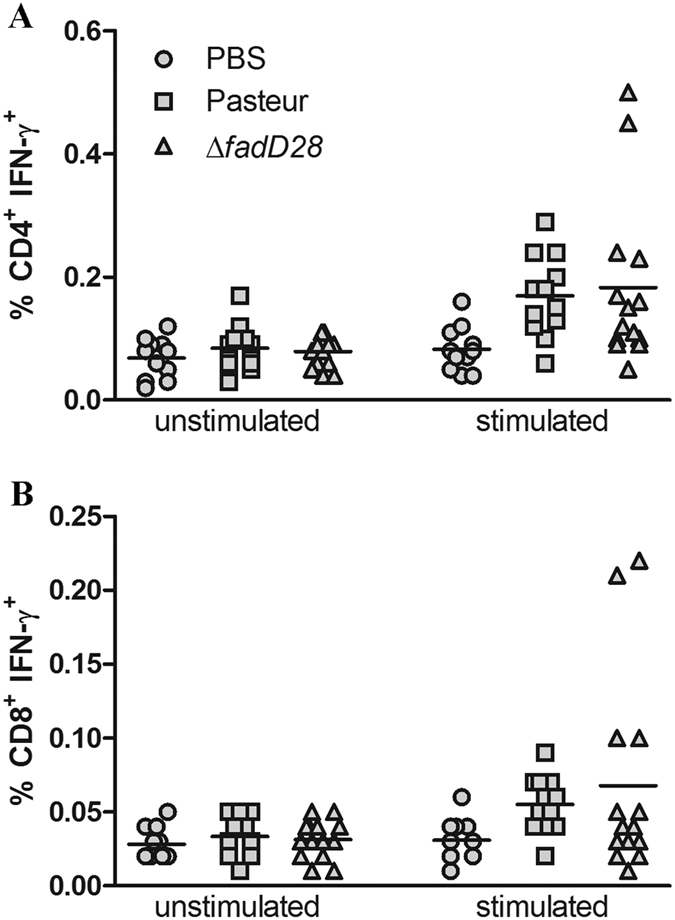
The loss of PDIMs/PGLs does not affect production of IFN-γ. Intracellular cytokine staining analysis of IFN-γ production by (**A**) CD4^+^ and (**B**) CD8^+^ T-cells. C57BL/6 mice were immunized subcutaneously with the WT BCG-Pasteur, ∆*fadD28*, or PBS/0.01% Tween 80. At 9 weeks post-vaccination, mice were sacrificed and splenocytes were harvested. Splenocytes were incubated with or without PPD for 24 hr followed by staining for T-cell surface markers (CD3-PE, CD4-FITC, CD8a-PercyPCy5.5) and intracellular IFN-γ (IFN-γ-APC). Samples were analyzed by BD FACSCalibur^TM^ and FlowJo^©^ Software. Pooled results from two independent experiments; each data point represents one mouse.

To assess if the loss of PDIMs/PGLs affects the immunogenicity of BCG-Pasteur, we examined production of IFN-γ in vaccinated C57BL/6 mice. Currently, there is no proven immunological correlate of protection or “biomarker” for efficacy^34^,^35^,^36^, however BCG has been shown to induce a T helper cell 1 (Th1) type response that is characterized by the production of IFN-γ from CD4^+^ T cells^37^. A critical role of IFN-γ in the control of TB has been demonstrated in mice^38^,^39^ and humans^40^,^41^. As such, antigen specific IFN-γ produced by CD4^+^ T cells has been used most widely as a measure of protective immunity, even though IFN-γ alone is insufficient for protection against TB^42^. Thus, to examine the role of PDIMs/PGLs in immunogenicity of BCG, we used a C57BL/6 immunocompetent mouse model and measured antigen (PPD) specific IFN-γ production from both CD4^+^ and CD8^+^ T cells by intracellular cytokine staining. Interestingly, we found that the loss of PDIMs/PGLs in BCG-Pasteur did not significantly alter the amount of IFN-γ production from both CD4^+^ and CD8^+^ T cells, where comparable levels were observed between the WT- and ∆*fadD28*-vaccinated groups (Fig. 3, Supplementary Fig. S3). Detection of IFN-γ production by ELISA also yielded similar results (Supplementary Fig. S4). Levels of additional Th1 markers, IL-2 and TNF, were also similar between the WT- and ∆*fadD28*-vaccinated mice after PPD stimulation (Supplementary Fig. S5). Taken together, our results indicate that loss of PDIMs/PGLs does not affect BCG immunogenicity.

### Loss of PDIMs/PGLs reduces BCG-mediated protection against *M. tb*

To determine if the PDIM/PGL-deficient mutant of BCG-Pasteur retained the same capacity to protect against *M. tb*, we used an aerosol challenge model in BALB/c mice. Immunocompetent inbred mice (BALB/c and C57BL/6) are widely used for TB vaccine studies because of the low cost and the availability of immunological reagents^43^. Groups of mice were vaccinated subcutaneously with ~10^5^ CFU of BCG strains (BCG-Pasteur, ∆*fadD28*, ∆*fadD28* + pFADD28, BCG-Japan) or PBS as a control. BCG-Japan was included in this experiment for comparison because it is naturally deficient in PDIMs/PGLs^19^. At 8 weeks post-vaccination, the mice were aerogenically challenged with 400–600 CFU of *M. tb* H37Rv and bacterial burden in the lung and spleen was determined at 5 and 9 weeks post-challenge.

**Figure 4:**
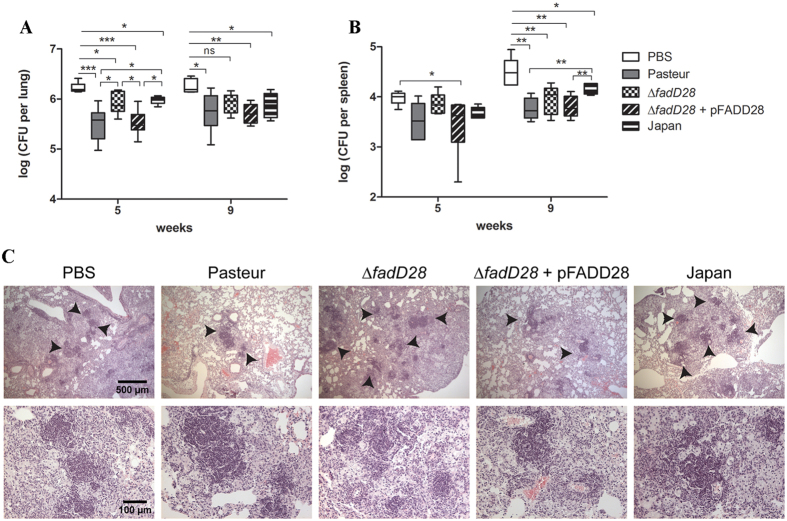
Loss of PDIMs/PGLs reduces BCG-mediated protection against *M. tb* in mice. BALB/c mice were vaccinated subcutaneously with ~10^5^ CFU of the BCG strains or PBS as a control. At 8-weeks post vaccination, mice were aerogenically challenged with *M. tb*. Mice were sacrificed at 5 and 9 weeks post-challenge and organs were examined for bacterial burden and pathology. The *M. tb* burden in the (**A**) lungs and (**B**) spleen was shown (6 mice per group per time point; data were plotted as box-whiskers in which the whiskers represent the minimum and maximum of all data. *p < 0.05; **p < 0.01; ***p < 0.001; one-way ANOVA, Tukey’s post hoc test). (**C**) Histological analysis of lung sections from mice in each group at 9 weeks post-challenge. Samples are stained with H&E. Arrows indicate regions of granuloma-like lesions. Top row is 50x magnification; bottom row is 200x magnification.

At week 5 post-challenge, the non-vaccinated group of BALB/c mice had a mean *M. tb* burden of 6.23 log_10_ CFU in the lungs (Fig. 4A). Mice vaccinated with WT BCG-Pasteur, ∆*fadD28*, the complemented strain, and BCG-Japan had on average 5.49 log_10_, 5.93 log_10_, 5.51 log_10_, and 5.98 log_10_ CFU of *M. tb* in the lungs, respectively. Compared to the PBS group, mice vaccinated with BCG strains had significantly lower *M. tb* burdens, with a reduction of 0.3 log_10_ (∆*fadD28*, BCG-Japan) and 0.7 log_10_ CFU (BCG-Pasteur, the complemented strain). Interestingly, mice vaccinated with ∆*fadD28* or BCG-Japan had significantly higher *M. tb* burdens than those vaccinated with WT BCG-Pasteur or the complemented strain, by ~0.45 log_10_ CFU (*p* < 0.05, one-way ANOVA, Tukey’s post hoc test).

At week 9 post-challenge, mice vaccinated with BCG-Pasteur, the complemented strain or BCG-Japan had significantly lower *M. tb* burdens in the lungs compared to the PBS control by 0.41 log_10_ (*p* < 0.05), 0.52 log_10_ (*p* < 0.01), and 0.34 log_10_ (*p* < 0.05), respectively (Fig. 4A, one-way ANOVA, Tukey’s post hoc test). The difference between the ∆*fadD28* and the PBS groups was not statistically significant nor was the difference between the WT and the ∆*fadD28* groups.

The aerosol infection model best mimics natural infection, and the levels of *M. tb* disseminated to the spleen are anticipated to be much lower than in the lungs^43^. Consistently, our data showed that the amounts of *M. tb* disseminated to the spleen were lower than that in the lungs by ~1.0–2.0 log_10_ CFU (Fig. 4B). At week 5 post-infection, mice vaccinated with BCG-Pasteur and the complemented strain had 0.35 and 0.38 log_10_ CFU fewer *M. tb* in the spleen than the PBS control group, respectively (Fig. 4B). There was no difference in the *M. tb* burden between mice vaccinated with ∆*fadD28* and the PBS control (3.91 log_10_ vs. 3.98 log_10_ CFU). At week 9, compared to the PBS group, mice vaccinated with BCG-Pasteur (ΔCFU = 0.77 log_10_, *p* < 0.01), ∆*fadD28* (ΔCFU = 0.55 log_10_, *p* < 0.01), the complemented strain (ΔCFU = 0.73 log_10_, *p* < 0.01), or BCG-Japan (ΔCFU = 0.37 log_10_, *p* < 0.05) had significantly lower *M. tb* burdens in the spleen. Mice vaccinated with BCG-Pasteur or the complemented strain had significantly lower *M. tb* burdens than those vaccinated with BCG-Japan (*p* < 0.01, one-way ANOVA, Tukey’s post hoc test). Mice vaccinated with ∆*fadD28* appeared to have higher *M. tb* burdens than those vaccinated with BCG-Pasteur or the complemented strain, but the differences were not statistically significant (Fig. 4B).

Histological analysis of *M. tb* challenged mice showed consistent differences in lung pathology between the different BCG-vaccinated cohorts. Vaccination with WT BCG-Pasteur or the complemented strain appeared to reduce the number of granuloma-like lesions in the lung, whereas mice vaccinated with the BCG-Pasteur ∆*fadD28* strain or BCG-Japan had lung pathology similar to the PBS control group (Fig. 4C).

C57BL/6 and BALB/c mice are highly resistant to *M. tb* infection and do not form caseous granulomas in the lungs that are typical of human TB disease^43^,^44^. This represents a major limitation of the murine model for vaccine studies. Alternatively, the guinea pig model is considered a more stringent test of vaccine efficacy^45^. Guinea pigs are highly susceptible to *M. tb* infection and develop clinically relevant symptoms, including weight loss and decreased pulmonary function due to extensive pulmonary infiltration. A recent study comparing vaccine testing from three different laboratories highlighted the reliability and reproducibility of the guinea pig model in obtaining efficacy data^46^.

**Figure 5:**
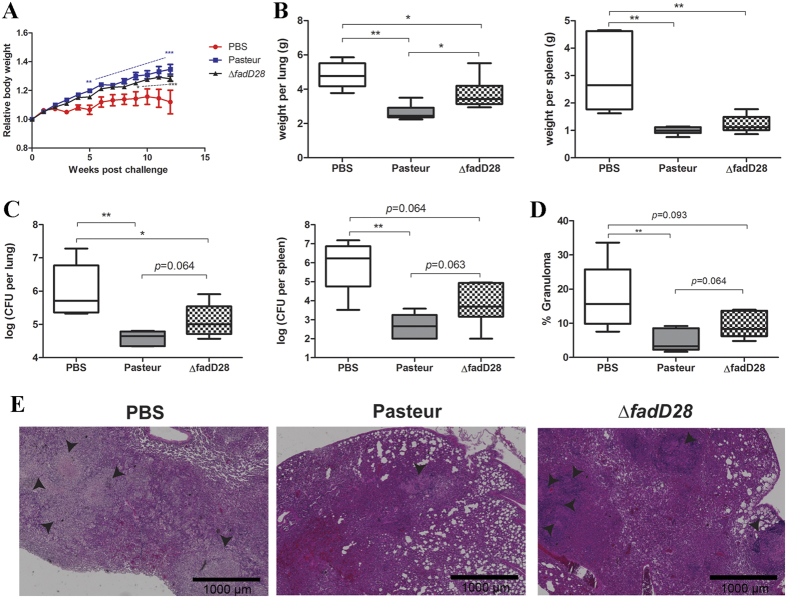
Loss of PDIMs/PGLs reduces BCG-mediated protection against *M. tb* in guinea pigs. Guinea pigs were vaccinated subcutaneously with WT BCG-Pasteur, ∆*fadD28* or PBS. At 10 weeks post-vaccination, guinea pigs were aerogenically challenged with *M. tb*. (**A**) Relative body weight (normalized to the body weight at time of *M. tb* challenge) of guinea pigs post infection. Data are plotted as mean ± SEM (*n*=6). **p < 0.01; ***p < 0.001, two-way ANOVA. (**B**) Lung and spleen weights of guinea pigs at 12-weeks post challenge. Data are plotted as box-whiskers in which the whiskers represent the minimum and maximum of all data (*n*=6). *p < 0.05; **p < 0.01, Mann-Whitney test. (**C**) *M. tb* burden in the lungs and spleen plotted as box-whiskers (*n*=6). **p < 0.01, Mann-Whitney test. (**D**) Quantitation of lung area affected by granuloma (% of total lung area). Six slides from each group were analyzed and data are plotted as box-whiskers. **p < 0.01, Mann-Whitney test. (**E**) Histological analysis of lung sections from guinea pigs in each group. Samples are stained with HE. Arrows indicate regions of granuloma-like lesions (40x magnification). Scale bar represents 1000 μm.

To examine if the reduced protection of the PDIM/PGL-deficient mutant of BCG-Pasteur against *M. tb* can also be observed in the guinea pig model, we conducted a *M. tb* challenge experiment in Hartley guinea pigs. Groups of six guinea pigs were vaccinated subcutaneously with WT BCG-Pasteur, ∆*fadD28*, or PBS and were aerogenically challenged 10 weeks later with *M. tb*. Prior to infection, guinea pigs of all groups exhibited similar weight gain. After challenge, one guinea pig in the PBS control group reached the humane end-points (loss of 20% maximal body weight and/or labored/heavy breathing) and was euthanized at week 10. Beginning at week 5 post-challenge, guinea pigs vaccinated with WT BCG-Pasteur gained significantly more weight than the PBS group (Fig. 5A). Guinea pigs vaccinated with ∆*fadD28* also showed significant weight gain compared to the PBS group starting at week 9 (Fig. 5A). The experiment was terminated at week 12 post-challenge and the lungs and spleen of these animals were isolated for further analyses. Mean guinea pig lung weights in the PBS and Δ*fadD28* groups were 4.81 g and 3.71 g, respectively. In contrast, the mean lung weight of WT BCG-Pasteur vaccinated guinea pigs was 2.62 g, which was significantly lower than those vaccinated with Δ*fadD28* or PBS (*p* < 0.05 and *p* < 0.01, Mann-Whitney test, Fig. 5B). Guinea pig spleen weights followed a similar trend, where the mean spleen weights in the PBS, Δ*fadD28*, and WT groups were 3.08, 1.22, and 0.99 g, respectively, although the difference between Δ*fadD28* and WT groups were not statistically significant (Fig. 5B).

Mean CFU counts of *M. tb* in the lungs of the PBS, Δ*fadD28*, and WT groups were 6.57 log_10_, 5.33 log_10_ and 4.63 log_10_, respectively (Fig. 5C). Notably, guinea pigs vaccinated with Δ*fadD28* had 0.7 log_10_ CFU more *M. tb* than those vaccinated with WT BCG-Pasteur and the difference was approaching significance (*p* = 0.064, Mann-Whitney test, Fig. 5C). The *M. tb* burden in the spleen exhibited a similar trend, where guinea pigs vaccinated with Δ*fadD28* had ~1.5 log_10_ more *M. tb* counts than those vaccinated with WT BCG-Pasteur (*p* = 0.063, Mann-Whitney test, Fig. 5C).

Histological examination of the lungs in the unvaccinated guinea pig group showed numerous granulomatous lesions with occasionally visible central necrosis (Fig. 5D,E), and large areas of the lungs were affected (Fig. 5D, mean = 18.02%). Lungs obtained from guinea pigs vaccinated with WT BCG-Pasteur or ∆*fadD28* had smaller, primarily non-necrotic granuloma-like lesions (Fig. 5D,E). Importantly, the affected area of the lungs from WT-vaccinated guinea pigs (mean = 4.68%) was smaller than that from ∆*fadD28*-vaccinated guinea pigs (mean = 9.29%) (Fig. 5D, *p* = 0.064, Mann-Whitney test). This difference is consistent with our observations that the bacterial burden in the lungs obtained from WT-vaccinated guinea pigs was reduced compared to that from ∆*fadD28*-vaccinated guinea pigs (Fig. 5C). Taken together, our results from both mouse and guinea pig models suggest that the loss of PDIMs/PGLs compromises the ability of the BCG vaccine to protect against *M. tb* infection.

## Discussion

BCG was derived from a virulent strain of *M. bovis* through *in vitro* attenuation (230 passages) from 1908 to1921. Beginning in 1924, BCG was distributed to various countries worldwide, resulting in a number of genetically distinct substrains. The mechanisms of BCG attenuation remain incompletely understood^17^. The loss of RD1, which encodes the type VII secretion system ESX-1, contributes to the attenuation of BCG^14^,^15^,^47^. However, recombinant BCG strains complemented with RD1 only partially restored the virulence, suggesting additional mechanisms are involved^31^,^48^. Comparative genome analyses revealed a number of genetic polymorphisms including deletions, duplications, and SNPs in BCG strains^9^,^11^,^12^,^13^,^14^. Some of these genetic changes are shared by subgroups of BCG strains whereas others are specific to individual strains. Despite the vast number of publications on BCG, studies to evaluate the impact of genetic polymorphisms on BCG vaccine properties (safety and efficacy) have been scarce. BCG strains distributed after 1927 (i.e. late BCG strains) contains an additional deletion of RD2, which encompasses genes *Rv1978* to *Rv1988* and includes important antigens such as MPT62 (encoded by *Rv1980c*)^15^. Deletion of RD2 may have attenuated the virulence of late BCG strains since a RD2-deletion mutant of *M. tb* H37Rv was more attenuated than the parental strain^49^. However, recombinant BCG-Pasteur complemented with *Rv1979c-Rv1982* did not improve protection against pulmonary TB, although it reduced the dissemination of *M. tb* to the spleen^50^. Late BCG strains also contain a point mutation in *mmaA3*, which impairs the production of methoxymycolate. However, complementation of a late BCG strain (BCG-Danish) with wild type *mmaA3*, which restored the production of methoxymycolate, had no effect on the virulence of BCG and its effect on protection was not determined^51^. The lack of experimental and clinical evidence demonstrating the impact of genetic differences among BCG has led to the argument that strain variation is not a significant factor for BCG effectiveness^18^. In this study, we demonstrated that the loss of PDIMs/PGLs, which occurs naturally in a subset of BCG strains, had a significant effect on the safety and protective efficacy of BCG, providing evidence that differences in BCG strains can influence vaccine effectiveness. A randomized trial study comparing two BCG strain in 300,000 infants in Hong Kong found that BCG-Pasteur, administered at a lower dosage, provided a significantly greater (40%) protection against childhood forms of TB than BCG-Glaxo^52^. Based on the finding of current study, the loss of PDIMs/PGLs in BCG-Glaxo^19^ is likely an important factor affecting its efficacy.

The loss of PDIMs/PGLs in BCG-Japan, -Moreau and -Glaxo correlates with their superior safety records in clinical studies over other BCG strains^17^,^19^,^53^. However, the existence of other mutations that distinguish BCG strains precludes a simple comparison of PDIM/PGL producers and non-producers to determine the importance, if any, of PDIMs/PGLs for vaccine safety and protection. To address this question, we constructed a PDIM/PGL-deficient strain from BCG-Pasteur, a PDIM/PGL producer, and performed a comparative study of the isogenic strains.

Consistent with the well-established role of PDIMs/PGLs in mycobacterial virulence, the loss of PDIMs/PGLs reduced the virulence of BCG-Pasteur, as demonstrated in the SCID mouse model. The ability of BCG-Pasteur to replicate in SCID mice and to cause morbidity was compromised when the production of PDIMs/PGLs was abrogated (Fig. 2). However, the loss of PDIMs/PGLs did not affect the immunogenicity of BCG-Pasteur, specifically its ability to induce antigen-specific IFN-γ production by CD4^+^ and CD8^+^ T cells (Fig. 3). Unexpectedly, the loss of PDIMs/PGLs also decreased the efficacy of BCG against *M. tb* challenge. This was demonstrated in both mouse and guinea pig models. As classically demonstrated^54^,^55^,^56^, the *M. tb* infection of BALB/c or C57BL/6 mice by aerosol challenge is followed by two phases. The progressive phase, in which *M. tb* grows essentially uninhibitedly for the first 3–4 weeks, results in 6–7 log_10_ CFU in the lungs. This is followed by the stationary phase in which further *M. tb* growth is inhibited by adaptive immunity. We found that at week 5 post-challenge, the *M. tb* burden in the lungs of BALB/c mice vaccinated with WT BCG-Pasteur was significantly lower than those vaccinated with the ∆*fadD28* strain (Fig. 4). However, this difference diminished at the stationary phase of infection (week 9 post-challenge) presumably because mice have begun to control the *M. tb* infection at this time. Considering the drawbacks of the mouse model (e.g., highly resistant to *M. tb* infection), we moved to the guinea pig model, which allows the evaluation of a broader spectrum of disease phenotypes. Although the change in body weight was not sensitive enough to distinguish between guinea pigs vaccinated with different BCG strains, as demonstrated previously^57^, the lung weights of guinea pigs vaccinated with ∆*fadD28* was on average 41.6% higher than those vaccinated with WT BCG-Pasteur (Fig. 5). The difference in spleen weight between these two groups was less significant presumably because the *M. tb* burden in the spleen was much lower than in the lungs as a result of the aerosol challenge route. Increased organ weights (lungs and spleen) have been associated with more severe disease phenotypes and frequently observed in guinea pigs infected with virulent *M. tb*^58^,^59^. Consistently, the *M. tb* burden in ∆*fadD28*-vaccinated guinea pigs was higher than those vaccinated with WT BCG-Pasteur, by 0.7 log_10_ and 1.5 log_10_ CFU in the lungs and spleen, respectively (Fig. 5). Collectively, these data provide strong evidence that the loss of PDIMs/PGLs reduces the protective efficacy of BCG.

Our finding has practical implications for the clinical preparations of BCG vaccines. The natural loss of PDIMs/PGLs in BCG-Japan, -Moreau, and -Glaxo likely occurred randomly during *in vitro* passaging. This is consistent with several observations. Firstly, independent mutations in biosynthetic genes account for the defective biosynthesis of PDIMs/PGLs in these BCG-strains. BCG-Japan contains a frame-shift single nucleotide insertion within *ppsA*^60^, and BCG-Moreau contains a deletion that disrupts both *fadD26* and *ppsA*^12^. The genetic mutation responsible for the loss of PDIMs/PGLs in BCG-Glaxo has yet to be identified. Secondly, spontaneous loss of PDIMs has been frequently observed in *M. tb* H37Rv (which produces PDIMs but not PGLs) during *in vitro* experiments^61^,^62^,^63^. Because this event occurs at such a high frequency, it is necessary to confirm the presence of PDIMs in all parental strains and recombinant clones before undertaking *in vivo* virulence studies^49^,^63^. Spontaneous loss of PDIMs in BCG-Pasteur during *in vitro* passage has also been reported^64^. Moreover, the clinical preparations of BCG-Japan actually contain two subpopulations, one producing PDIMs/PGLs and the other defective in PDIMs/PGLs^60^,^65^. Considering the high selective pressure under *in vitro* conditions for PDIM/PGL-negative clones, and our finding that the loss of PDIMs/PGLs has a significant impact on BCG vaccine safety and efficacy, it is essential that quality control programs in BCG manufacturers should include a regular test of PDIMs/PGLs of vaccine preparations.

The PDIM/PGL-deficient strain is less virulent but also less protective, suggesting a positive correlation between virulence and efficacy. Consistent with this notion, a previous study found that recombinant BCG strains complemented with the RD1 region exhibited increased virulence in SCID mice but also better protection in C57BL/6 mice and guinea pigs^31^,^48^. More recently, a comparative analysis of the virulence and efficacy of 13 different BCG strains in SCID and BALB/c mice, respectively, also revealed a general trend that more virulent BCG strains were also more effective in protection against *M. tb* challenge^66^. Currently, the strategies for developing the next generation of TB vaccines include live vaccines (recombinant BCG or attenuated *M. tb*) and subunit vaccines^67^,^68^. The lack of protective efficacy of MVA85A, the most advanced subunit vaccine candidate thus far, in a recent clinical trial study^69^ further underlines the importance of live vaccine research^70^. The positive correlation between virulence and efficacy we observed suggests that when developing recombinant BCG or attenuated *M. tb*, there needs to be a fine balance between these two factors in order to achieve optimal protection while maintaining an acceptable level of safety.

## Materials and Methods

### Bacterial strains and culture conditions

*Mycobacterium bovis* BCG strains, BCG-Pasteur and BCG-Japan, were grown at 37 °C in Middlebrook 7H9 broth (Difco^™^) supplemented with 0.2% glycerol, 10% albumin-dextrose-catalase (ADC; BD BBL^™^), and 0.05% Tween80 or on Middlebrook 7H11 agar (Difco^™^) supplemented with 0.5% glycerol and 10% oleic acid-albumin-dextrose-catalase (OADC; BD BBL^™^). *Escherichia coli* strain DH5α was used for routine manipulation and propagation of plasmid DNA. *E. coli* DH5α was grown in LB broth or agar (BioShop). Antibiotics were added as required: kanamycin, 50 μg/ml for *E. coli* and 25 μg/ml for BCG; hygromycin, 150 μg/ml for *E. coli* and 75 μg/ml for BCG.

### Generation of a PDIM/PGL-deficient mutant of BCG-Pasteur

Specialized phage transduction was used to generate a PDIM/PGL-deficient mutant of BCG-Pasteur as described previously^71^. Briefly, the allelic exchange construct was made by amplifying upstream and downstream regions flanking the *fadD28* gene from BCG-Pasteur genomic DNA using the primer sets 5′-ACTAGTGATTTCGACACTCGGTAA-3′ (SpeI)/5′-AAGCTTGTCTTCTTTGAAGGT-3′ (HindIII) and 5′-TCTAGAGATTTTCACGCCTTT-3′(Xba1)/ 5′-GGTACCAGTTCGATA ATG G-3′ (KpnI), respectively (restriction sites are underlined). The upstream amplicon was digested and ligated into a SpeI/HindIII-digested pJSC284 cosmid, containing a hygromycin resistance marker (hyg^R^). The resulting vector was then digested with XbaI and KpnI and ligated to the downstream amplicon, creating the complete allelic exchange construct. Correct insertion of both amplicons was confirmed by PCR using locus-specific primers. The recombinant construct was cloned into a conditionally replicating TM4 shuttle phasmid, phLR, and specialized transducing mycobacteriophage were generated by electroporating *M. smegmatis* mc^2^155 at the permissive temperature (30 °C). Putative knockout mutants were obtained by transducing BCG-Pasteur at the non-permissive temperature (37 °C) and selecting hygromycin-resistant colonies. Deletion of *fadD28* was confirmed by Southern blot (Amersham) analysis using a 500 bp probe against the upstream region of *fadD28*, generated with primers 5′-TCCAACCTCGTCTCAGCT-3′ and 5′-CGCCAT GGGTCCACCA-3′, following the manufacturer’s protocol. The complementation plasmid was generated by amplifying a 2094 bp fragment containing a wild type (WT) copy of *fadD28*, using the forward primer 5′-GGTACCAAGCCAGTTAGGGGC-3′ (KpnI) and reverse primer 5′-AAGCTTCAGTCCG GGGAGGAC-3′ (HindIII), and cloned into a KpnI/HindIII-digested pME shuttle vector to generate pFADD28. Three to five clones of each strain were tested.

### Lipid analysis by thin layer chromatography

Production of PDIMs/PGLs was examined using two-dimensional thin layer chromatography (2D-TLC), according to published procedures^19^,^72^. Briefly, the apolar lipid fraction was extracted from 50 mg (dry weight) of BCG and analyzed on silica gel 60 plates (EMD Chemicals Inc.). For detection of PDIMs, apolar lipids were developed with petroleum ether/ethyl acetate (98:2, 3×) in the first dimension and petroleum ether/acetone (98:2) in the second dimension. Lipids were visualized by staining plates with 5% phosphomolybdic acid followed by gentle charring. For detection of PGLs, the apolar lipid extract was developed with chloroform/methanol (96:4, v/v) in the first dimension and toluene/acetone (80:20, v/v) in the second dimension, followed by charring with α-naphthol. The productions of PDIMs/PGLs were periodically checked to ensure strain integrity.

### Ethics statement

All of the animal procedures were approved by the University of Toronto Animal Care Committee. All experimental procedures were performed in accordance with the Canadian Council on Animal Care (CCAC) and University of Toronto regulations.

### Analysis of BCG virulence in SCID mice

Female Fox Chase CB17^®^ SCID mice were purchased from Charles River Laboratories and the mice were age-matched (7–8 weeks) within each experiment. Mice (4–6 per group per time point) were infected intravenously via the tail vein with ~10^4^ or ~10^5^ CFU of the different BCG strains in 0.2 ml PBS/0.01% Tween80. At 1, 7, 21, 42, 52, and 79 days post-infection, the lungs were harvested, homogenized in PBS, and plated on 7H11 agar to enumerate bacterial burden. CFU was counted after incubation at 37 °C for 3 weeks. Bacterial counts from lung homogenates harvested at day 1 post-infection were used as an indicator of initial infection dose. These experiments were done in duplicate.

### Immunogenicity studies

Female C57BL/6 mice were purchased from Charles River Laboratories and were age-matched (6 weeks) within each experiment. Four to nine mice per group were inoculated subcutaneously on the scruff of the neck with approximately ~10^4^ CFU in 0.2 ml PBS/0.01%Tween80 of parental BCG-Pasteur or the *fadD28* knockout strain. Control mice were given 0.2 ml of PBS/0.01% Tween80. After 9 weeks, mice were euthanized, splenocytes were isolated, and intracellular IFNγ was measured. Briefly, splenocytes were seeded at 2×10^6^ cells/well in 100 μl in triplicate and stimulated with 2.5 μg/well of purified protein derivative (PPD) (Statens Serum Institute, Denmark) or complete RPMI (cRPMI; RPMI/10% FBS/1% L-glutamine/1% penicillin/streptomycin) as a control and incubated at 37 °C and 5% CO_2_. After 19 hours of stimulation, GolgiPlug (BD Biosciences) was added in a 1:1000 dilution and incubated for an additional 5 hours. After a total of 24 hours stimulation, plates were centrifuged and the cell pellet was washed in 200 μl FACS Buffer (0.5% BSA/PBS) and incubated with Fc Block (eBiosciences) diluted in FACS Buffer (1:400) for 15 minutes. The cells were then washed with FACS Buffer and stained for extracellular T-cell surface markers: CD3-PE, CD4-FITC, and CD8a-PercyPCy5.5 (BD Biosciences) diluted in FACS Buffer for 30 minutes. Following extracellular marker staining, the cells were permeabilized and fixed with 1×CytoFix/CytoPerm (BD Biosciences) for 20 minutes. Cells were then washed with 1×PermWash (BD Biosciences) and incubated with IFNγ-APC (BD Biosciences) for 30 minutes to stain for intracellular IFNγ. Immediately following staining, cells were analyzed on a BD FACSCalibur^TM^ flow cytometer (BD Biosciences). A total of 300,000 events per sample were collected in the lymphocyte gate and analyzed using FlowJo V7.6. Gates for analysis were set based on isotype controls. These experiments were done in duplicate.

### Protection against *M. tb* challenge

Mouse model: Groups of 13–15 female BALB/c mice (Charles River Laboratories) were vaccinated subcutaneously on the scruff of the neck with ~10^5^ CFU of the BCG strains in 0.2 ml PBS/0.01% Tween80 or PBS/0.01% Tween80 alone as a control. At 8 weeks post-vaccination, mice were aerogenically challenged with 400–600 CFU of *M. tb* H37Rv using a GlasCol nebulizer. Mice were euthanized at 5 and 9 weeks post-challenge (6–7 mice per group per time point) to harvest the lungs and spleen. A portion of the organs were fixed in 10% formalin for histological analysis. The remaining portion was homogenized and plated on 7H11 agar to enumerate burden of *M. tb* in the lung and spleen. Plates were incubated at 37 °C and counted after 2.5–3 weeks. These experiments were done in duplicate.

Guinea pig model: Groups of six female Hartley guinea pigs (Charles River Laboratories) were vaccinated subcutaneously with 5 × 10^4^ CFU of parental BCG-Pasteur or the *fadD28* knockout strain in 0.2 ml PBS/0.01% Tween80 or PBS/0.01% Tween80 alone as a control. At 10 weeks post-vaccination, guinea pigs were infected with ~1000 CFU of *M. tb* H37Rv by an aerosol challenge using a GlasCol nebulizer. At 12 weeks post-challenge, guinea pigs were euthanized to obtain the lungs and spleen. A portion of the spleen and the caudal lobe of the left lung were fixed in 10% formalin for histological analysis. The remaining portion of the spleen and the entire right lung lobes were homogenized separately and plated on 7H11 agar to quantify the *M. tb* burden in the lungs and spleen. Colonies were counted after incubation at 37 °C for three weeks. This experiment was performed once.

### Histological analysis

Fixed tissues were embedded into paraffin blocks at the Centre for Modeling Human Disease (Toronto Centre for Phenogenomics). Serial sections (4 μm thick for mouse tissues and 5 μm thick for guinea pig tissues) were prepared and kept at 37 °C for more than 12 hours. The sections were deparafinized in three changes of xylene for 3 minutes each and rehydrated in four consecutive washes of alcohol (100%, 100%, 95%, and 70%) for 3 minutes each. Sections were stained with hematoxylin and eosin (EMD Chemicals) or Acid Fast staining kit (Surgipath) according to standard procedures and were examined using a Leica microscope (Life Technologies) or Cytation^TM^ 5 (BioTek). Perceived areas of granulomatous lesions were determined by ImageJ.

### Statistical Analysis

Majority of the dataset passed the Kolmogorov–Smirnov normality test. One-Way Analysis of Variance (One-way ANOVA) with Tukey’s multiple comparisons were performed for *M. tb* burdens (log_10_ transformed CFU data) when there are more than 3 groups. Two-Way ANOVA were performed on data (CFU or body weight) of 3 or more groups at multiple time points. Student’s *t* test (Mann-Whitney test) was performed when there are 3 or fewer groups.

## Additional Information

**How to cite this article**: Tran, V. *et al.* Loss of Lipid Virulence Factors Reduces the Efficacy of the BCG Vaccine. *Sci. Rep.* **6**, 29076; doi: 10.1038/srep29076 (2016).

## Supplementary Material

Supplementary Information
