# Supplementary material for: Loss of Lipid Virulence Factors Reduces the Efficacy of the BCG Vaccine
Source: Sci Rep. 2016 Jun 30;6:29076. doi: 10.1038/srep29076 (PMC4928182; doi:10.1038/srep29076)
Supplement: Supplementary Information [file srep29076-s1.pdf]

Loss of Lipid Virulence Factors Reduces the Efficacy of the BCG Vaccine

Vanessa Tran[†], Sang Kyun Ahn, Mark Ng, Ming Li, and Jun Liu[*]

Department of Molecular Genetics, University of Toronto, Toronto, ON, Canada

*Corresponding author: Jun Liu (jun.liu@utoronto.ca)

[†]Present address: Vanessa Tran, Sandra A. Rotman Laboratories, Sandra Rotman Centre for Global Health, Toronto General Research Institute – University Health Network, Toronto, ON, Canada

Supplementary Information

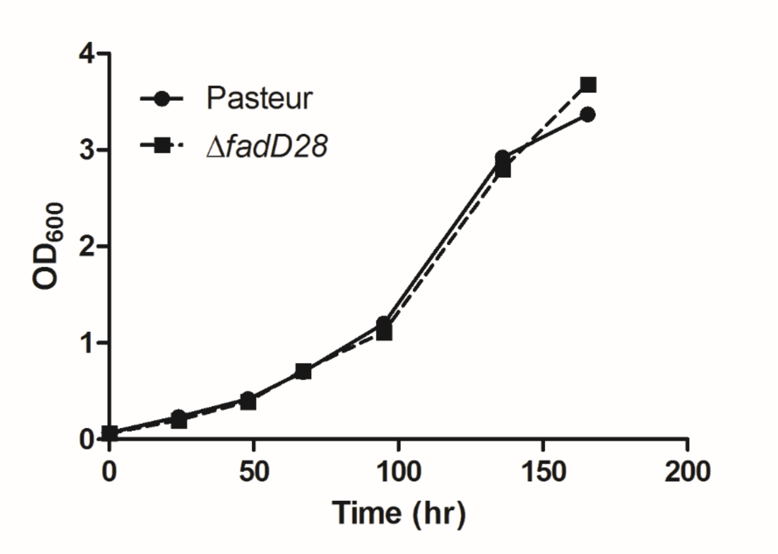

Fig S1. Growth curve of WT and PDIM/PGL knockout strains of BCG-Pasteur. Strains were grown in 7H9 broth and OD_{600} was measured every 24 hr.

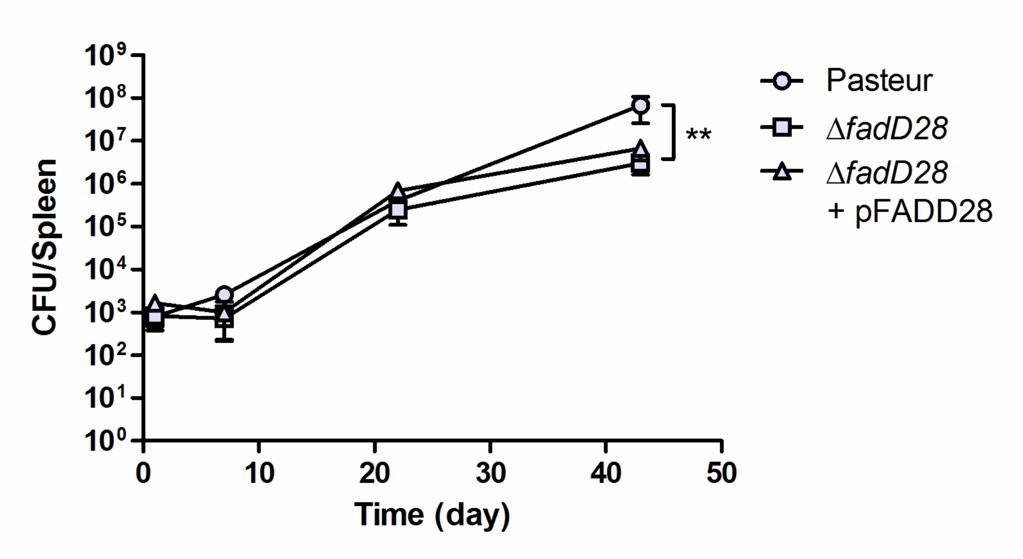

Fig S2. The PDIM/PGL deficient mutant of BCG-Pasteur is less virulent in SCID mice.

SCID mice were infected intravenously with 10^4 CFU of BCG-Pasteur (Pasteur), the Δ*fadD28*, or complemented strain (Δ*fadD28* + pFADD28). Bacterial burden in the spleen was determined at various time points (**, p<0.01, BCG-Pasteur vs. Δ*fadD28*; two-way ANOVA).

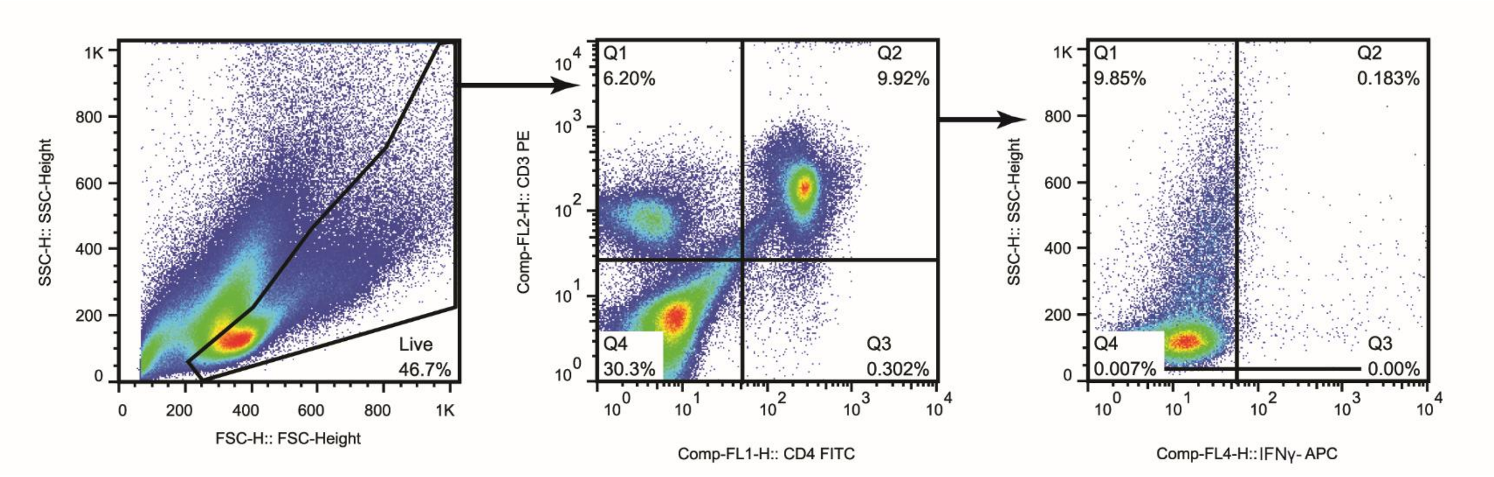

Fig S3. Sample gating strategy for detection of IFN-γ-producing T-cells. The population of live cells were gated from the lymphocyte population. Live cells were subsequently plotted with $CD3^+$ vs $CD4^+$/$CD8^+$ and double positive cells were further plotted with SSC vs IFN-γ. Gates were set based on isotype controls.

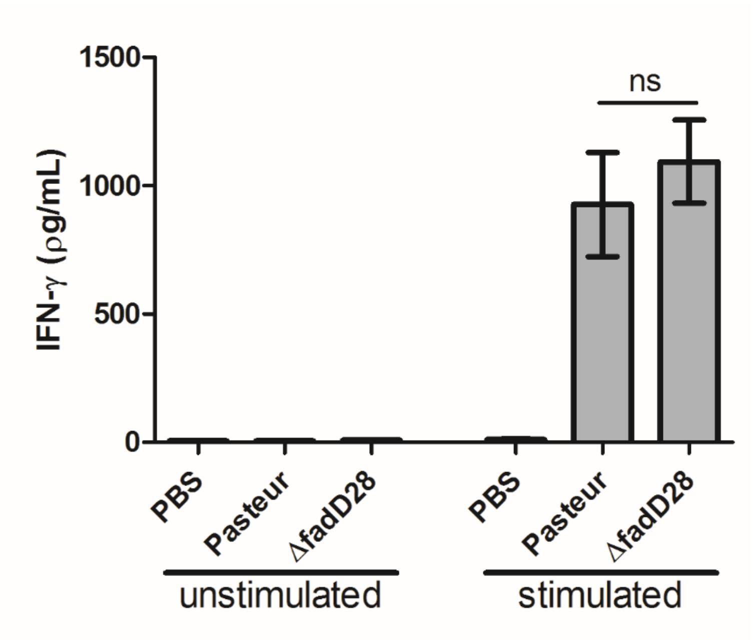

Fig S4. The loss of PDIMs/PGLs does not affect production of IFN-γ. C57BL/6 mice were immunized subcutaneously with the WT BCG-Pasteur, Δ*fadD28*, or PBS/0.01% Tween 80. At 9 weeks post-vaccination, mice were sacrificed and splenocytes were harvested. Splenocytes were incubated with or without PPD for 72 hr and IFN-γ was measured by ELISA (BD Biosciences) (ns, not significant).

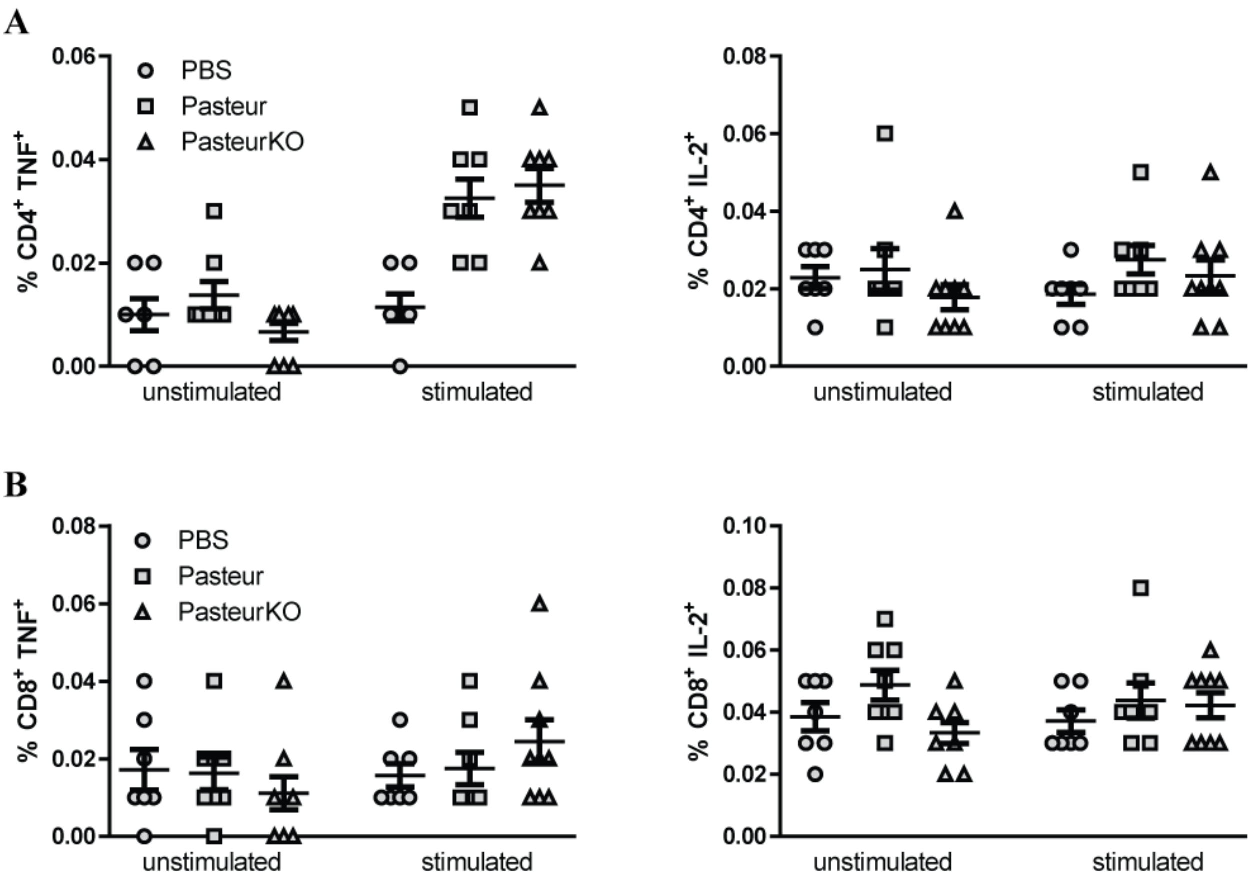

Fig S5. The loss of PDIMs/PGLs does not affect production of TNF or IL-2. Intracellular cytokine staining analysis of TNF and IL-2 production by $CD4^+$ and $CD8^+$ T-cells. C57BL/6 mice were immunized subcutaneously with the WT BCG-Pasteur, Δ*fadD28*, or PBS/0.01% Tween 80. At 9 weeks post-vaccination, mice were sacrificed and splenocytes were harvested. Splenocytes were incubated with or without PPD for 24 hr followed by staining for T-cell surface markers (CD3-PE, CD4-FITC, CD8a-PercyPCy5.5) and intracellular TNF and IL-2. Samples were analyzed by BD FACSCalibur™ and FlowJo© Software (mean ± SEM).
